# Diagnosis and Management of Beckwith-Wiedemann Syndrome

**DOI:** 10.3389/fped.2019.00562

**Published:** 2020-01-21

**Authors:** Kathleen H. Wang, Jonida Kupa, Kelly A. Duffy, Jennifer M. Kalish

**Affiliations:** ^1^Division of Human Genetics, Children's Hospital of Philadelphia, Philadelphia, PA, United States; ^2^Department of Pediatrics, Perelman School of Medicine, University of Pennsylvania, Philadelphia, PA, United States; ^3^Department of Genetics, Perelman School of Medicine, University of Pennsylvania, Philadelphia, PA, United States

**Keywords:** Beckwith-Wiedemann syndrome, methylation, diagnostic testing, mosaicism, cancer predisposition, tumor screening

## Abstract

Beckwith-Wiedemann syndrome (BWS) is a human genomic imprinting disorder that presents with a wide spectrum of clinical features including overgrowth, abdominal wall defects, macroglossia, neonatal hypoglycemia, and predisposition to embryonal tumors. It is associated with genetic and epigenetic changes on the chromosome 11p15 region, which includes two imprinting control regions. Here we review strategies for diagnosing and managing BWS and delineate commonly used genetic tests to establish a molecular diagnosis of BWS. Recommended first-line testing assesses DNA methylation and copy number variation of the BWS region. Tissue mosaicism can occur in patients with BWS, posing a challenge for genetic testing, and a negative test result does not exclude a diagnosis of BWS. Further testing should analyze additional tissue samples or employ techniques with higher diagnostic yield. Identifying the BWS molecular subtype is valuable for coordinating patient care because of the (epi)genotype-phenotype correlations, including different risks and types of embryonal tumors.

## Introduction

Beckwith-Wiedemann syndrome (BWS) is a human imprinting disorder that leads to overgrowth. It is associated with genetic and epigenetic changes on the chromosome 11p15 region (1), which includes imprinted genes that regulate fetal and postnatal growth. BWS is often diagnosed neonatally or in early childhood and has a broad clinical spectrum of features that vary in severity. These features include macroglossia, abdominal wall defects, lateralized overgrowth, enlarged abdominal organs, and an increased risk for developing embryonal tumors during early childhood (2). BWS is now considered a spectrum (BWSp) ranging from classic BWS to isolated lateralized overgrowth (2, 3). BWS has an estimated prevalence of 1 affected child in 10,340 live births (4), with an increased risk associated with assisted reproductive technologies (ART) of around 1 in 1,100 (5–7).

To determine if molecular testing should be pursued and to establish a clinical diagnosis of BWS, a clinical scoring system is used (2). In this system, cardinal features include macroglossia, omphalocele, lateralized overgrowth, bilateral Wilms tumor, hyperinsulinism, and specific pathological findings such as adrenal cytomegaly or placental mesenchymal dysplasia. Each cardinal feature is given 2 points. Suggestive features include birthweight greater than two standard deviations above the mean, facial nevus simplex, polyhydramnios or placentomegaly, ear creases or pits, transient hypoglycemia, embryonal tumors, nephromegaly or hepatomegaly, and umbilical hernia or diastasis recti. Each suggestive feature is given 1 point. Patients with a clinical score ≥2 merit genetic testing for BWS. Patients with a score ≥4 based on cardinal and suggestive features satisfy a clinical diagnosis of classical BWS, even without molecular confirmation. Patients with a score <2 do not meet criteria for genetic testing. If a patient with a score ≥2 has negative genetic testing, alternative diagnoses and/or referral to a BWS expert should be considered. Genetic testing is also recommended for patients with a family history of BWS and a known heritable pathogenic 11p15 anomaly, which occurs in about 10–15% of patients (1).

BWS is a mosaic disorder and, as such, may warrant genetic testing on multiple tissues because a patient may have cells of different genetic or epigenetic compositions in their body (8). In the case of BWS, a patient may have some cells that carry the epigenetic/genetic change and some cells that do not. Mosaicism itself can also vary in severity within a patient as different tissues can have different ratios of affected to unaffected cells. Thus, molecular confirmation is not always possible due to tissue mosaicism. More details about testing are below under Molecular Genetic Testing for BWS.

## Molecular Mechanisms of BWS

**Figure 1:**
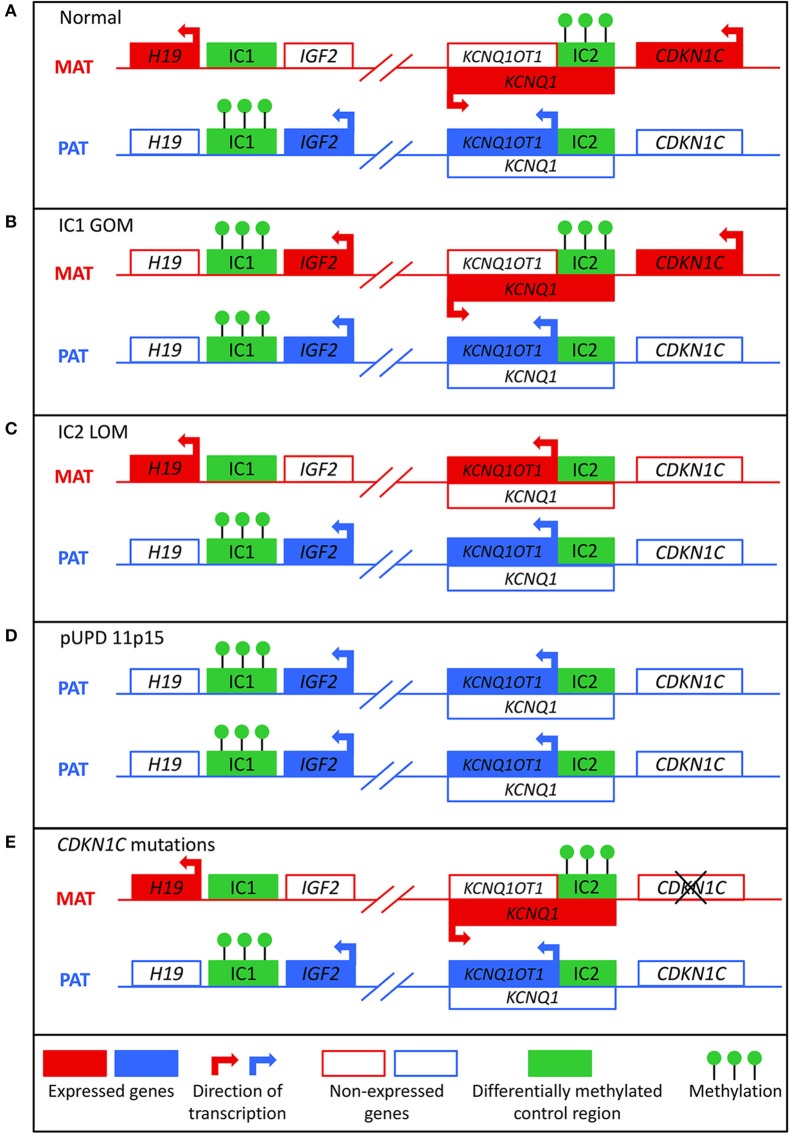
Molecular mechanisms that can lead to Beckwith-Wiedemann syndrome. **(A)** In normal cells, the paternal allele is methylated at imprinting control region 1 (IC1) and the maternal allele is methylated at imprinting control region 2 (IC2). **(B)** In IC1 gain of methylation (IC1 GOM), both the maternal and paternal alleles are methylated at IC1, which leads to downregulation of *H19* and overexpression of *IGF2*. **(C)** In IC2 loss of methylation (IC2 LOM), the maternal allele loses methylation at IC2, which leads to expression of *KCNQ1OT1* and downregulation of *CDKN1C*. **(D)** In paternal uniparental disomy of 11p15 (pUPD), there are two copies of the paternal chromosome, which leads to overexpression of *IGF2* and decreased expression of *CDKN1C*. **(E)** Mutations of the maternal *CDKN1C* gene can result in loss of function.

BWS involves molecular aberrations within a cluster of imprinted genes on the chromosome 11p15.5-11p15.4 region, as depicted in Figure 1. There are two functionally independent domains, the telomeric and the centromeric, each with its own imprinting control region (1). The telomeric domain includes the long non-coding RNA (lncRNA) *H19* and insulin-like growth factor 2 (*IGF2*). *H19* is a maternally expressed lncRNA in the embryo and placenta, but it is silenced in most tissues after birth except in cardiac and skeletal muscle (9, 10). It may have a role in both tumor formation and suppression (11). *IGF2* is a growth factor that is paternally expressed in the fetus and placenta, and biallelically expressed in the liver after birth (12). The telomeric domain is controlled by the *H19/IGF2* intergenic differentially methylated region (*H19/IGF2*:IG DMR), also known as imprinting control region 1 (IC1) that is located 2 kilobases upstream of *H19* (1). Shared enhancers of *H19* and *IGF2* and a CTCF binding factor-dependent insulator located between the two genes control imprinting of this locus (13, 14). In mouse, CTCF binds to the imprinting control region (ICR) on the maternal allele to produce an insulator that results in expression of *H19* and silencing of *Igf2* (15). On the paternal allele, methylation of the ICR prevents CTCF binding, which leads to *Igf2* expression and silencing of the *H19* promoter. Similar mechanisms of regulation occur in humans, but the IC1 region is much larger in humans (16).

The centromeric domain includes *KCNQ1*, the regulatory lncRNA *KCNQ1OT1*, and *CDKN1C*, a cell cycle inhibitor. *KCNQ1* encodes a voltage-gated potassium channel, and it is initially maternally expressed during early embryogenesis but becomes biallelically expressed during fetal development (17). *KNCQ1OT1* is a paternally expressed lncRNA transcribed antisense to *KCNQ1* (18). *CDKN1C* encodes a G1 cyclin-dependent kinase inhibitor, and it negatively regulates cell growth and proliferation. It is expressed in the embryo and placenta as well as postnatally in the body (19). The centromeric domain is controlled by the *KCNQ1OT1* transcription start site differentially methylated region (*KCNQ1OT1*:TSS DMR), also known as imprinting control region 2 (IC2) that is located on the 5′ end of *KCNQ1OT1* and includes the promoter region for *KCNQ1* (20). In mice, the maternal allele is methylated so that *Kcnq1ot1* is not expressed and *Kcnq1* and *Cdkn1c* are expressed. On the paternal allele, the *Kcnq1ot1* promoter is not methylated so the lncRNA is expressed and *Kcnq1* and *Cdkn1c* are silenced (21). Regulation of this ICR seems to be similar in mice and humans (16).

About 80% of patients with BWS have a known molecular defect in the 11p15 region, most commonly due to aberrant DNA methylation (1). Normally, the paternal allele is methylated at IC1 and the maternal allele is methylated at IC2 (Figure 1A). These methylation marks are established in the germline and must be maintained throughout the reprogramming that occurs post-fertilization in the zygote (22). Gain of methylation at IC1 on the maternal allele (IC1 GOM) is found in about 5–10% of patients. This leads to downregulation of *H19* and expression of *IGF2* on the maternal allele (Figure 1B) (23). Loss of methylation at IC2 on the maternal allele (IC2 LOM) is found in about 50% of patients (24). This leads to derepression of *KCNQ1OT1* and downregulation of *CDKN1C* on the maternal allele (Figure 1C) (23, 25). Paternal uniparental isodisomy (pUPD) occurs when a patient has two copies of the paternal chromosome 11p15 and none of the maternal, and this occurs in about 20% of patients (24). The extent of the disomy can range from segmental to genome wide, but with regards to the 11p15 region, pUPD leads to overexpression of *IGF2* and decreased *CDKN1C* expression (Figure 1D) (26). pUPD can be caused by errors in meiosis I or meiosis II in the gametes, or more frequently in BWS by postzygotic errors in mitotic recombination during early embryogenesis (27, 28). *CDKN1C* mutations on the maternal allele occur in about 5% of sporadic cases and 40% of familial BWS (Figure 1E) (24). If the mutation is maternally inherited, there is a 50% chance recurrence risk with variable expressivity (29). Chromosomal abnormalities including duplications, deletions, and translocations of the 11p15 region are found in <5% of patients (24). These alterations are usually inherited in an autosomal dominant manner, and the recurrence risk depends on the sex of the parent carrying the affected allele (1).

There is a higher frequency of multiple gestations in patients with BWS compared to the general population (30). The majority of cases are monozygotic female twins with discordant features where one twin is more affected by BWS than the other (30), but there is great variability in the degree of phenotypic discordance (31). It is hypothesized that an epigenetic event causing BWS occurs prior to, and perhaps triggers the twinning process (30, 32, 33). The affected cells diffuse among the embryos in a multiple pregnancy, resulting in a mosaic distribution. Cohen et al. (31) present this theory of “diffused mosaicism” where the timing of the embryologic twinning relative to the timing of the epigenetic event likely influences the degree of BWS affectedness and degree of mosaicism.

Assisted reproductive technologies (ART), such as *in vitro* fertilization (IVF) and intracytoplasmic sperm inject (ICSI) may impact the establishment and/or the maintenance of DNA methylation at imprinted loci (7, 34). There is a 10-fold increased risk of BWS with ART and an absolute risk of about 1 in 1,100 (5). More than 90% of children with BWS conceived by ART have IC2 LOM (5). Further research is needed to illuminate the relationship between ART and imprinting defects.

## Molecular Genetic Testing for BWS

### Overview of Genetic Testing Strategies

Mosaicism can pose a significant challenge to genetic testing in BWS because different tissues may have different proportions of affected BWS cells (8). First-line diagnostic testing is usually performed on DNA derived from blood-leukocytes (2). Other samples such as buccal swabs, skin fibroblasts, or cells of mesenchymal origins including surgical resections and/or excisions of hyperplastic tissues, can improve the detection of mosaicism (3, 35, 36). A negative result does not exclude a diagnosis of BWS and may be the result of low-level mosaicism that is below the level of detection, a rare balanced chromosomal rearrangement, or another currently unrecognized cause (2). In up to 20% of patients with a BWS phenotype, a molecular diagnosis remains unknown (1). This may be due to tissue mosaicism, as testing multiple tissues improves diagnostic yield (3). Patients without a confirmed molecular diagnosis should be evaluated for clinical features suggestive of different diagnosis and appropriate additional testing should be considered (2). If other features are not present and the clinical score is ≥4, the patient may have classical BWS without molecular confirmation (2). Patients with a score <4 and with isolated lateralized overgrowth may still be part of the BWSp (3).

**Figure 2:**
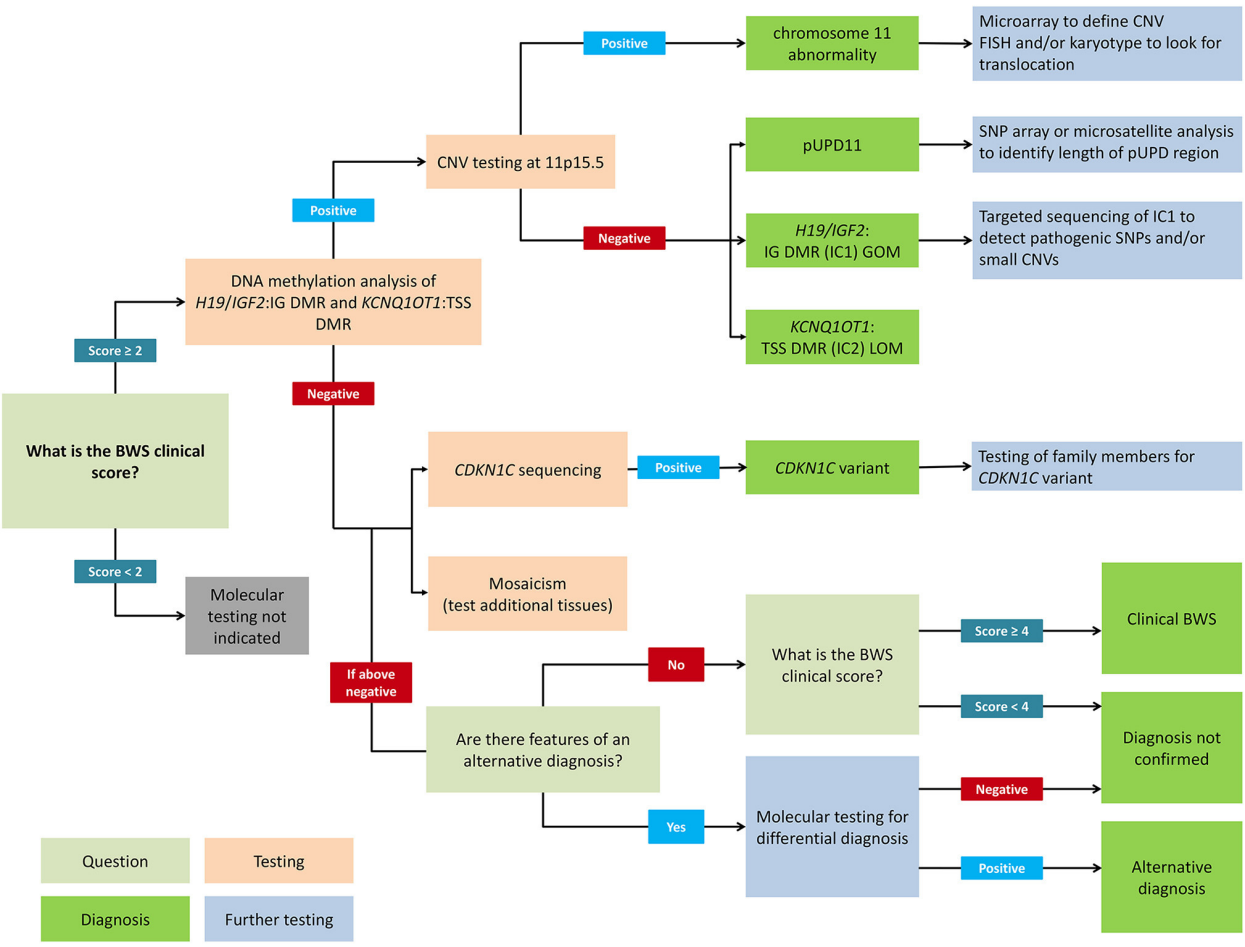
Flowchart for molecular diagnosis of Beckwith-Wiedemann syndrome. Patients with BWS clinical score ≥2 should receive genetic testing while patients with clinical score <2 do not meet the criteria for testing. Recommended first-line testing (highlighted in orange) should analyze methylation at *H19/IGF2*:IG DMR (IC1) and *KCNQ1OT1*:TSS DMR (IC2) and copy number variation (CNV). These tests can yield positive molecular diagnoses of chromosome 11 abnormalities, paternal uniparental disomy of chromosome 11 (pUPD), IC1 gain of methylation (IC1 GOM), and IC2 loss of methylation (IC2 LOM) (highlighted in dark green). Further testing (highlighted in blue) can determine chromosomal abnormalities more precisely. If DNA methylation testing is negative, *CDKN1C* sequencing is recommended, or additional tests for rare chromosomal translocations. Negative test results can also be due to tissue mosaicism, and additional tissue samples can be tested. Differential diagnoses should also be considered, but patients with clinical score ≥4 can have a clinical diagnosis of BWS even without molecular confirmation. Adapted from Brioude et al. (2).

Figure 2 presents a flowchart for how to approach a molecular diagnosis for BWS. First-line testing procedures should determine the IC1 and IC2 methylation level and the differentially methylated region (DMR) copy number (2). Abnormal methylation is present in IC1 GOM, IC2 LOM, pUPD11 (which shows both IC1 GOM and IC2 LOM), and in copy number variations (CNVs) (35). Methylation-specific multiplex ligation-dependent probe amplification (MS-MLPA) is currently the most common diagnostic test because it simultaneously detects percent methylation and DMR copy number status (37, 38). However, other methylation-specific techniques are more sensitive to low-level mosaicism, and multiple tissues should be tested in patients with low-level mosaicism (36, 39, 40). To confirm pUPD, chromosomal microarray analysis (CMA) such as a single nucleotide polymorphism (SNP) based microarray analysis can detect low-level mosaicism and determine the length of the region of pUPD, which may impact care (27, 41, 42). Genome-wide pUPD (GWpUPD), or mosaic paternal isodisomy, may affect up to 10% of patients with pUPD of chromosome 11p15 and involves additional clinical features and elevated tumor risk (26, 43). *CDKN1C* mutations are detected through gene sequencing, and detection of a pathogenic variant allows for cascade testing of family members to clarify recurrence risk (29, 44).

If a CNV is detected, chromosome microarray is recommended to determine the size and nature of the deletion or duplication (41, 45). Karyotyping or fluorescence *in situ* hybridization (FISH) can also be used to identify chromosomal translocations depending on the nature of the breakpoints (46). In patients with IC1 GOM, up to 20% may have small CNVs in the DMR, which are associated with a high risk of recurrence (47, 48). These can sometimes be detected using MS-MLPA but require targeted IC1 sequencing, especially if there is a family history of BWS (49, 50). Deletions in IC2 are rare (45), and there is currently no specific recommendation to analyze CNVs in patients with IC2 LOM (2). About one third of IC2 LOM patients have been reported to have a multilocus imprinting disturbance (MLID), but the clinical significance is uncertain so testing is usually not indicated (51).

For BWS patients who are part of a multiple pregnancy, knowing the zygosity and chorionicity is important for appropriate diagnosis. For dizygotic dichorionic gestations, no evaluation is indicated for the unaffected twin but for monozygotic monochorionic and dichorionic gestations, the twin should receive a clinical examination by a geneticist (31). To accurately diagnose discordant monozygotic twins, buccal swab is the preferred source of DNA because DNA from blood cells or saliva may show aberrant methylation in an unaffected twin due to shared circulation during fetal development (52).

### Diagnostic Tests for Aberrant Methylation

The most widespread diagnostic test is methylation-specific multiplex ligation-dependent probe amplification (MS-MLPA) because it can detect both DMR methylation and copy number variation (37, 38). However, it cannot determine the size or content of CNVs, and other tests such as CMA or FISH analyses are more suitable for this (37). MS-MLPA is a polymerase chain reaction (PCR)-based method and uses multiple test probes in the 11p15 region and in other loci across the genome (37, 38). Some of the probes for the 11p15 region are methylation specific and contain the Hha I restriction enzyme site within a CpG island. After the probes and DNA incubate together, the sample is divided into two aliquots, one for traditional MLPA and the other for methylation-specific (MS) analysis. The first aliquot for traditional MLPA uses the ligation of two half-probes to detect CNVs. The second aliquot for MS analysis includes the addition of Hha I restriction enzyme, which specifically targets unmethylated sequences for degradation so only methylated samples are amplified. The relative amounts of the target DNA sequence are quantified by fluorescently-labeled primers, which are used to calculate the methylation index (37, 38). MS-MLPA can also identify the parent of origin of small genomic duplications and deletions (38), but further testing such as a CMA or FISH is recommended. It is important to have a wide cohort of controls and to match analyses with controls from the same tissue (36). Using a single experiment, MS-MLPA can detect CNV and methylation status within the 11p15 region with high specificity and reliability (45). Furthermore, this test can be performed efficiently and at low costs with a small quantity of DNA, making it an ideal first-line diagnostic test (37, 38).

To more precisely study low-level mosaicism, other methylation-sensitive PCR methods are needed. In quantitative methylation-sensitive PCR, genomic DNA is treated with sodium bisulfite then amplified with quantitative TaqMan PCR (39, 40). The bisulfite treatment deaminates unmethylated cytosines to uracil while methylated cytosines are preserved. The TaqMan probes are labeled with different fluorophores to discriminate between methylated and unmethylated DNA, and the methylation index is calculated based on the fluorescence intensity from each allele. Using allele-specific methylated multiplex real-time quantitative PCR (ASMM RTQ-PCR), Azzi et al. (40) identified a range of normal methylation smaller than that of MS-MLPA, meaning the assay is more sensitive and is able to detect minute changes in methylation. Another method is methylation-specific high-resolution melting (MS-HRM) that uses differences in melting profile of methylated and unmethylated DNA to detect methylation (53). Following bisulfite treatment and PCR amplification, a fluorescent intercalating dye is added to the DNA, and the change in fluorescence is monitored as the DNA melts. The unmethylated allele will begin melting at a lower temperature followed by the methylated allele. MS-HRM has similar results and limitations as MS-MLPA and other methylation-specific PCR techniques (53).

### Diagnostic Tests for Uniparental Disomy and Chromosomal Abnormalities

Chromosomal microarray analysis (CMA), FISH, and karyotyping can detect copy number variations, chromosomal abnormalities, and can confirm uniparental isodisomy (46). CMA is the most common microarray technology used to identify deletions and duplications, but it provides limited information on the structural rearrangements. Either FISH or karyotyping is usually performed with CMA to confirm results and to identify translocations and insertions (46).

There are two major microarray analyses: comparative genomic hybridization (CGH) and single nucleotide polymorphism (SNP) analysis. Both microarrays detect submicroscopic changes that are too small to be detected through conventional karyotyping by comparing hybridization intensities between the patient sample and normal reference DNA (54). Through this approach, labeled patient DNA and labeled reference DNA compete to hybridize to normal metaphase-arrested human DNA. An equal distribution of the hybridized patient and reference DNA is indicative of a healthy sample, whereas an imbalanced ratio is indicative of chromosomal aberrations in the patient DNA (54). CGH can identify deletions or duplications of a few kilobases in size whereas SNP probes can identify variations at a single site in DNA when present (55). A major limitation of CGH is its inability to detect balanced chromosomal rearrangements, or UPD and low-level mosaicism (46, 54).

SNP microarrays can simultaneously detect gain/loss CNVs and copy-neutral loss of heterozygosity, as well as the parent of origin of CNVs to detect uniparental disomy (27, 45, 56). MS-MLPA and methylation-sensitive PCR methods can detect pUPD indirectly when both IC1 and IC2 have abnormal methylation, but SNP microarrays can quantitatively determine the size of UPD based on the extent of the SNPs affected (26, 36, 41). SNP microarrays can detect uniparental disomy when both alleles in the patient come from a single parent. SNP microarrays are the most sensitive method for UPD and the associated mosaicism (41), and can distinguish low-level mosaicism (1–5%) from normal samples using B-allele frequencies (BAF) (27, 42). They are also able to distinguish UPD from chromosomal abnormalities more precisely than karyotyping or FISH (42). Furthermore, a genome-wide SNP array can be utilized to distinguish mosaicism from chimerism, which occurs when there are two different cell lines with two complete sets of DNA within the body (27). Microsatellite arrays, which analyze highly polymorphic short tandem repeats (STRs) in the DNA, are similar to SNP microarrays in that both can detect UPD, but SNP microarrays are more sensitive (41).

While microarrays identify changes at specific regions of the genome, karyotyping identifies larger chromosome differences. Karyotyping detects structural changes >3–10 Mb in size and it is well-suited for complex rearrangements involving multiple chromosomes (46).

Fluorescence *in situ* hybridization (FISH) can detect structural changes of genes with higher resolution than karyotyping (57). FISH is a hybridization technique that uses fluorescent probes to bind to specific DNA sequences with high specificity in order to detect the presence or absence of these stretches of DNA (58). Prior knowledge of the abnormal region is required to design FISH probes, and only a few probes can be used at a time (58). While karyotyping and FISH can also distinguish chromosomal abnormalities from mosaic UPD, they cannot determine the size of a disomy nor the size of a chromosomal deletion or duplication (42). After identifying the chromosomal abnormalities in the patient, testing should be extended to other family members as appropriate (2).

### Diagnostic Tests for *CDKN1C* Mutations

To detect *CDKN1C* mutations or other gene mutations in the 11p15 region, genetic sequencing is performed. Briefly, PCR is performed to amplify the region of interest, then Sanger sequencing is used to query the sequence for candidate mutations.

### Prenatal Testing

BWS can be diagnosed molecularly in some prenatal cases, but due to mosaicism, a negative test result cannot exclude a diagnosis (23) and postnatal testing should also be performed to confirm results (59). Genetic counseling should include discussion of the benefits and limitations of each test offered. Testing is usually indicated by abnormal ultrasound, including omphalocele, macroglossia, or enlarged abdominal organs in the fetus. Placental mesenchymal dysplasia, polyhydramnios, or increased alpha-fetoprotein (AFP) in the second trimester can also occur (59). Positive family history mainly arises from *CDKN1C* mutations or chromosomal abnormalities such as deletions/duplications. Both native and cultured amniocytes can be used for testing, but cultured cells may show features that do not correlate with the true biological status of the fetus or placenta (59). If testing is undertaken, methylation testing and *CDKN1C* sequencing is recommended. Testing on chorionic villus sampling (CVS) can be limiting because of confined placental mosaicism, which might not reflect the (epi)genetic status of the fetus (60) and thereby CVS testing that is negative or showing low-level changes would require an amniocentesis and/or postnatal testing for further evaluation. Maternal contamination is also a possibility, so parallel microsatellite analysis of maternal and fetal short tandem repeats (STRs) is strongly advised (59). While a positive methylation or chromosomal test result confirms a diagnosis of BWS, a negative result cannot exclude a diagnosis. Due to the complexity and heterogeneity of BWS, tissue mosaicism and other molecular alterations could escape detection. Postnatal testing should be performed to confirm any results (59).

## Management and Care of Patients With BWS

**Figure 3:**
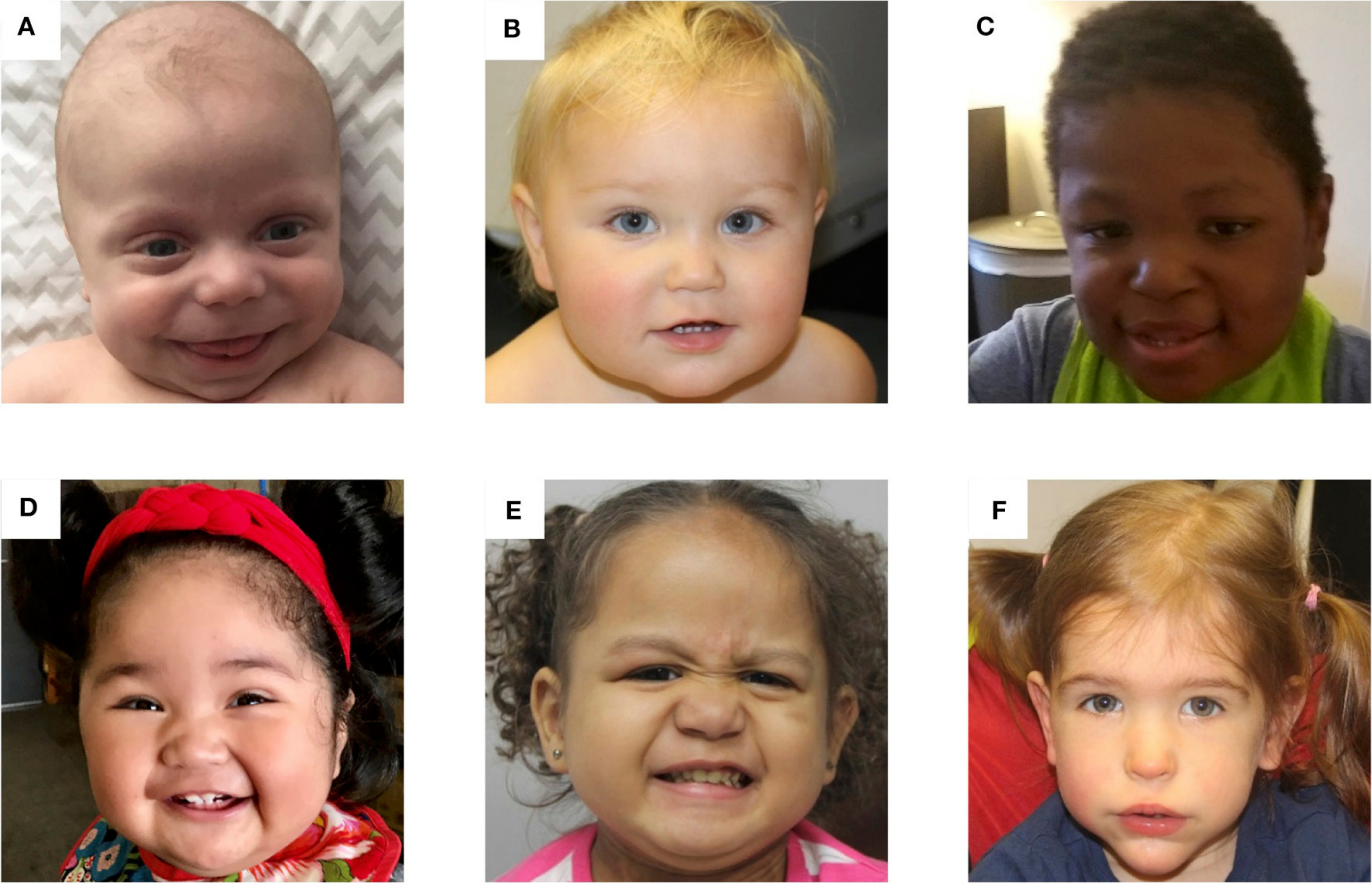
Photos of six patients with BWS due to **(A)** IC2 loss of methylation (IC2 LOM), **(B)** IC1 gain of methylation (IC1 GOM), **(C)** chromosomal rearrangements (deletions, duplications), **(D)** paternal uniparental isodisomy 11 (pUPD), **(E)** genome-wide paternal uniparental isodisomy (GWpUPD), and **(F)** *CDKN1C* mutation. Written consent was obtained from the parents of every participant to publish these identifying images.

Determining the molecular subtype of BWS is important because there are correlations between clinical phenotype and (epi)genotype (61–63). Figure 3 depicts facial photographs of patients with different molecular subtypes of BWS. IC1 GOM patients tend to have large birth weights, enlarged abdominal organs, and high incidence of tumors (28%) especially Wilms tumors, while IC2 LOM patients typically present with omphalocele, macroglossia, and nevus simplex, and have the lowest incidence of tumors (2.6%). UPD patients tend to have lateralized overgrowth and hyperinsulinism and an intermediate tumor incidence (16%). It is unknown if there is a correlation between the severity of the phenotype and the level of mosaicism or chromosomal isodisomy (61). Patients with *CDKN1C* mutations have similar features as those with IC2 LOM including omphalocele and nevus simplex, and they have an estimated tumor incidence of 6.9%, although limited data exists (62).

### Prenatal Care

For patients with a prenatal diagnosis, the management of individual congenital anomalies should follow standard protocols. BWS is associated with increased risk of polyhydramnios, gestational hypertension, pre-eclampsia, and preterm births (64–66), so appropriate arrangements for delivery and neonatal care should be made. Post-delivery complications may include neonatal hypoglycemia, respiratory obstruction due to macroglossia, and surgical repair of omphalocele (2).

### Hypoglycemia and Hyperinsulinism

Hypoglycemia occurs in about 30–60% of patients, and BWS-related neonatal hypoglycemia is due to excess insulin (62, 67, 68). It is generally transient and resolves within a few days, but in some cases persistent hyperinsulinism (HI) occurs, and therefore neonates with suspected BWSp should be screened for hypoglycemia before discharge (2). HI may require medical treatment such as diazoxide or somatostatin analogs such as octreotide and lanreotide, or in some cases subtotal pancreatectomy in HI that persists despite maximal medical therapies (68). Congenital HI is a rare condition seen most commonly in pUPD patients, and a diagnosis should be made with an endocrinologist who is familiar with this condition. HI in BWS can occur with or without mutations in the beta cell potassium channel genes (*ABCC8* and *KCNJ11*), which are also on chromosome 11p near the BWS region (68).

### Macroglossia

Macroglossia is seen in about 90% of patients with BWS (61, 62), and about 40% of children undergo a tongue reduction surgery (69). Need for surgery and timing of surgery depends on the clinical status of the patient. The most common indications for surgery include respiratory problems, obstructive sleep apnea, feeding difficulties, persistent drooling, problems with speech and articulation, and orthodontic problems (70, 71). An airway evaluation and polysomnography can provide further assessment for obstructive sleep apnea (72). If there are respiratory problems, surgery might need to be performed earlier in the neonatal period. Studies show that patients who receive surgery before 2–3 years tend to have good outcomes with favorable results including cosmetic improvement, adequate tongue mobility, and no substantial effect on taste (69, 71).

### Abdominal Wall Defects

For omphalocele and other abdominal wall defects, no specific recommendations have been given with regards to patients with BWS. The management of these features should follow standard protocols and local practices (2).

### Growth and Lateralized Overgrowth

Overgrowth in BWS occurs in about 43–65% of patients (61, 62), and lateralized overgrowth can occur when one side of the body is larger than the other. Postnatal growth is generally in the upper percentiles but slows down in late childhood (73). Lateralized overgrowth is the most frequent feature in pUPD patients (63), and the management will depend on the affected limbs. Leg length discrepancy (LLD) may require shoe lifts or surgical correction in some cases (74).

### Tumor Screening Protocols

BWS is a cancer predisposition syndrome with an overall tumor risk of about 8%, but each molecular subgroup is associated with a different tumor incidence and types of tumors (75, 76). The most common types of tumors are Wilms tumor (52%) and hepatoblastoma (14%), followed by neuroblastoma (10%), rhabdomyosarcoma (5%), and adrenal carcinoma (3%) (62). Cancer risk is the highest during the first 2 years of life and declines afterwards, and there is currently no evidence of an increased tumor risk in adults with BWS (2). IC1 GOM is associated with the highest incidence of tumor (28%), followed by pUPD (16%), *CDKN1C* mutation (6.9%), and the lowest with IC2 LOM (2.6%) (61, 62, 75). Patients with IC1 GOM are predisposed to Wilms tumor, which accounts for 95% of tumors in this group (62, 75), while patients with IC2 LOM are more likely to develop hepatoblastoma (77), and patients with *CKDN1C* mutations are predisposed to neuroblastoma (62, 75). Wilms tumor and hepatoblastoma occur with similar frequencies in patients with pUPD, and patients with GWpUPD seem to develop similar types of tumor as those with segmental pUPD but with an increased incidence of hepatic and/or adrenal tumors extending into young adulthood (78, 79). For patients with a clinical diagnosis of BWSp or negative molecular testing, there needs to be further research to understand their cancer risk (80).

Tumor screening protocols are recommended for earlier detection of tumors, reducing morbidity, and increasing patient survival. Guidelines developed by the American Association for Cancer Research Childhood Cancer Predisposition Workshop (AACR-CCPW) include full abdominal ultrasound (USS) every 3 months from diagnosis until the 4th birthday, renal ultrasound every 3 months from age 4–7 years, and AFP screening every 3 months until the 4th birthday for all patients with BWSp (81). Patients with *CDKN1C* mutations should also receive neuroblastoma screening, which includes urine VMA/HVA and chest X-ray every 3 months until the 6th birthday then every 6 months from age 6–10 years, in addition to abdominal imaging and AFP screening (82). In contrast Brioude et al. (2), an international consensus group consisting primarily of European experts, recommends abdominal ultrasounds every 3 months from diagnosis until the 7th birthday for the BWSp subgroups that are at the highest risk of cancer including IC1 GOM, pUPD, *CDKN1C* mutation, and other genomic rearrangements of the region and clinical BWS (2).

To screen for hepatoblastomas, measurements of serial serum alpha-fetoprotein (AFP) could lead to earlier detection than abdominal ultrasounds. However, interpreting AFP levels can be difficult during infancy and early childhood due to variable concentrations and wide range of normal values (83–85). The consensus group stated that AFP measurements should not be offered routinely because of the low incidence of hepatoblastoma in BWSp and the difficulties in interpretation (2).

AACR-CCPW identifies a 1% tumor risk threshold and therefore recommends abdominal USS and AFP screening for all subtypes of BWSp (81). While patients with IC2 LOM have an overall lower risk of tumor development, they have an increased risk of hepatoblastoma (77), which has significantly lower event-free survival rates compared to Wilms tumor or other embryonal tumors. Patients with localized and lower stage hepatoblastoma can achieve high survival rates between 80 and 100%, but patients with late stage tumors face a poorer prognosis (86, 87). Serum AFP levels to screen for hepatoblastoma should be interpreted in the context of the clinical picture, and patients with BWS tend to have higher AFP levels in early childhood compared to normal pediatric values (83, 84, 88). AFP levels are expected to decline over time and can be tracked with normograms (89), and large rises in AFP levels should be further investigated by repeat testing and additional imaging (81). AFP screening can be used to distinguish hepatoblastoma from infantile hepatic hemangioma, a benign vascular neoplasm (90, 91). Monitoring serial serum AFP levels can allow for early detection of hepatoblastoma, even before detection by abdominal imaging, which can lead to better patient outcomes (92, 93). Recently Mussa et al. (94) have developed a less invasive method for measuring AFP levels using dried blood spots that is as accurate as traditional venipuncture.

Children with BWS and Wilms tumor tend to have higher incidence of recurrence (95) and potential co-occurrence of progressive non-malignant renal diseases and bilateral Wilms tumor (96, 97). Multifocal or diffuse nephrogenic rests in one or both kidneys (nephroblastomatosis) are not easily distinguishable from Wilms tumor and may require MRI for diagnosis (97). Partial nephrectomy and nephron-sparing strategies for Wilms tumors are recommended if possible (96, 98). The presence of nephro-urological anomalies in patients with BWSp is 28–61% (99), including cortical and medullary cysts in about 10% of patients and higher incidence of hypercalciuria and nephrolithiasis (100). For adults with BWS, a detailed clinical review and renal ultrasound should be performed at 16 years to develop specific recommendations for surveillance for ongoing problems (2). There is no apparent association between BWSp and predisposition to common adult-onset carcinomas (101), but further research on adults with BWS is needed.

### Cardiac Features

Cardiac defects occur in up to 13–20% of patients with BWS, and there is higher incidence of congenital heart disease compared to the general pediatric population (61, 102, 103). Minor anatomical defects should be monitored by echocardiogram until spontaneous resolution, but more severe defects might require surgical correction (2). Although rare, patients with IC2 CNVs and/or genomic rearrangements of the region may be predisposed to long QT syndrome and require follow-up throughout adulthood (104, 105).

### Cognitive and Neurological Features

Patients with BWS usually have normal cognitive development, and a broader differential diagnosis should be considered in patients with an overgrowth disorder and learning disability without a 11p15 anomaly (2, 106). However, developmental delay can be associated with prematurity, severe hypoglycemia, unbalanced chromosomal rearrangements, and GWpUPD (78).

### Psychological Well-Being

The diagnosis of BWSp can affect patients and families psychologically and socially. Parents may be unprepared for the diagnosis because in many cases there is no relevant family history (2). The increased tumor risk and surveillance protocol can cause increased anxiety, but a survey of parents with children with BWS revealed that tumor screening decreases worry and is not burdensome (107). In children with macroglossia, some parents are worried that a large protruding tongue and persistent drooling may affect peer interactions and increase emotional difficulties (70, 108). Healthcare professionals should be aware of these psychosocial issues and refer families to specialists including genetic counselors, social workers, and psychologists, or offer support groups as appropriate (2).

## Discussion

BWSp is a complex multisystem disorder that can result from a variety of molecular changes in the 11p15 region. A range of different genetic diagnostic tests are used to detect aberrant methylation and chromosomal abnormalities, and the presented genetic testing strategies can guide clinicians when establishing a molecular diagnosis for BWSp. However, tissue mosaicism continues to pose a challenge to genetic testing, and a negative test result cannot exclude a diagnosis of BWSp. Even with negative molecular testing, a BWS clinical score ≥4 based on cardinal and suggestive features satisfies a clinical diagnosis of BWS. Nevertheless, identifying a molecular diagnosis is important in coordinating the care and management of patients with BWSp and testing multiple tissues if possible can improve molecular diagnosis.

## Author Contributions

KW performed the literature search and drafted the manuscript and some of the figures. JK performed a literature search and drafted the section on some of the testing techniques and made one of the figures. KD helped conceptualize the project and edited the manuscript. JMK conceptualized, organized, and edited the manuscript.

### Conflict of Interest

The authors declare that the research was conducted in the absence of any commercial or financial relationships that could be construed as a potential conflict of interest.
